# Supplementary material for: Identification of the Time Period during Which BMP Signaling Regulates Proliferation of Neural Progenitor Cells in Zebrafish
Source: Int J Mol Sci. 2023 Jan 15;24(2):1733. doi: 10.3390/ijms24021733 (PMC9863262; doi:10.3390/ijms24021733)
Supplement: Supplementary file 1 [file ijms-24-01733-s001.zip › ijms-2070311-supplementary.pdf]

Supplementary Figures and Legends

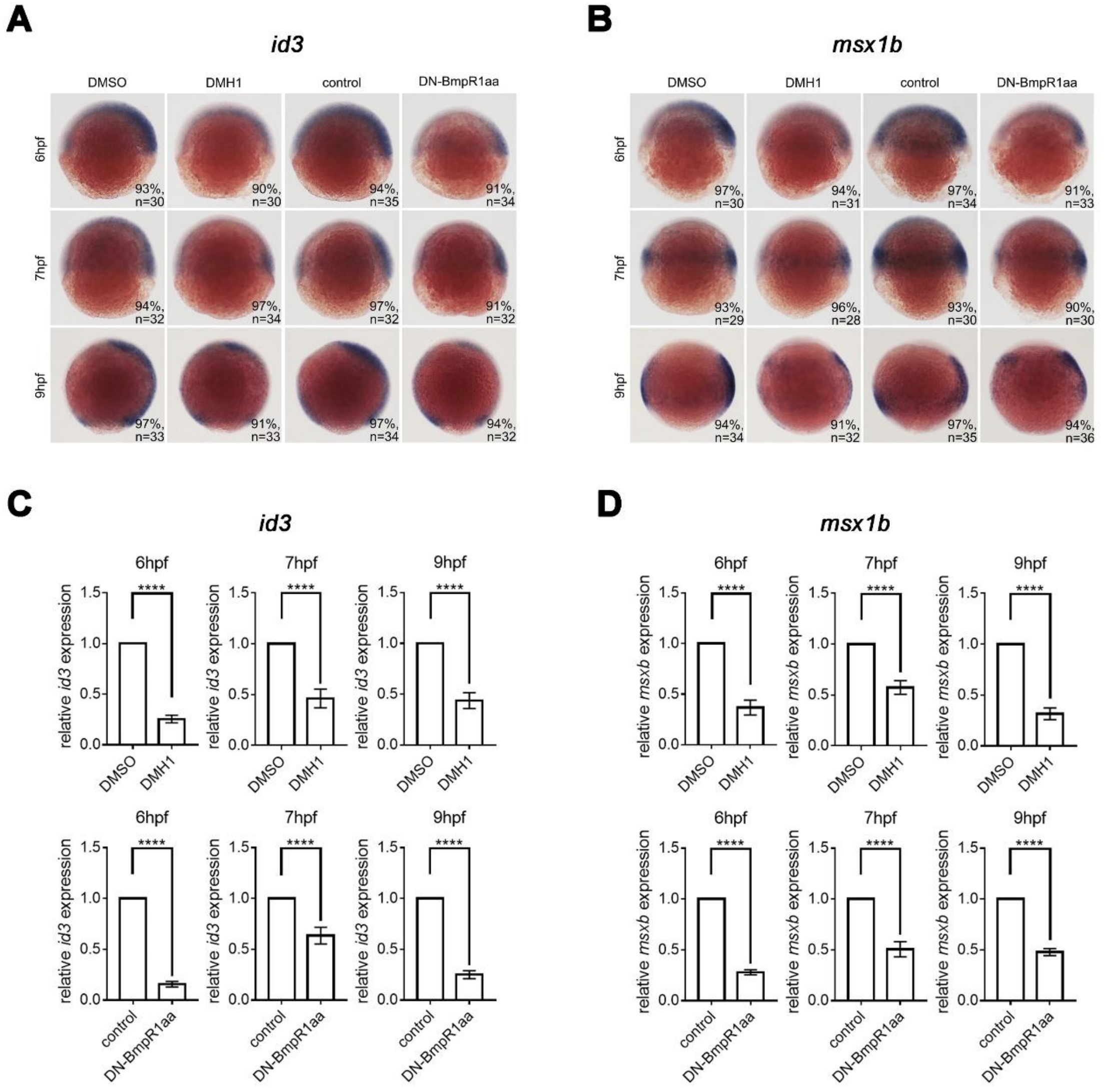

Figure S1. DN-Bmpr1aa and DMH1 are sufficient to inhibit the expression of *id3* and *msx1b*.

Photographs showing the results of *in situ* hybridization using *id3* and *msx1b* riboprobes with lateral views ventral to the left. At 5.3 hpf, *Tg (hsp70l:dnBmpr1aa-GFP)* embryos were heat-shock-treated, and wild-type embryos were treated with 0.5 μM DMH1. These embryos were raised and harvested at 6, 7, and 9 hpf for *in situ* hybridization, which showed that *id3* (A) and *msx1b* (B) expression was inhibited by DN-Bmpr1aa and DMH1. The expression levels of *id3* and *msx1b* in *in situ* hybridization in A and B were confirmed using qRT-PCR (C and D, respectively). The

percentages in each panel in A and B indicate the proportion of embryos displaying the same phenotype as that shown in the photographs of the total embryos examined. Quantitative data are presented as the mean ± standard deviation (SD); **** $p < 0.0001$.

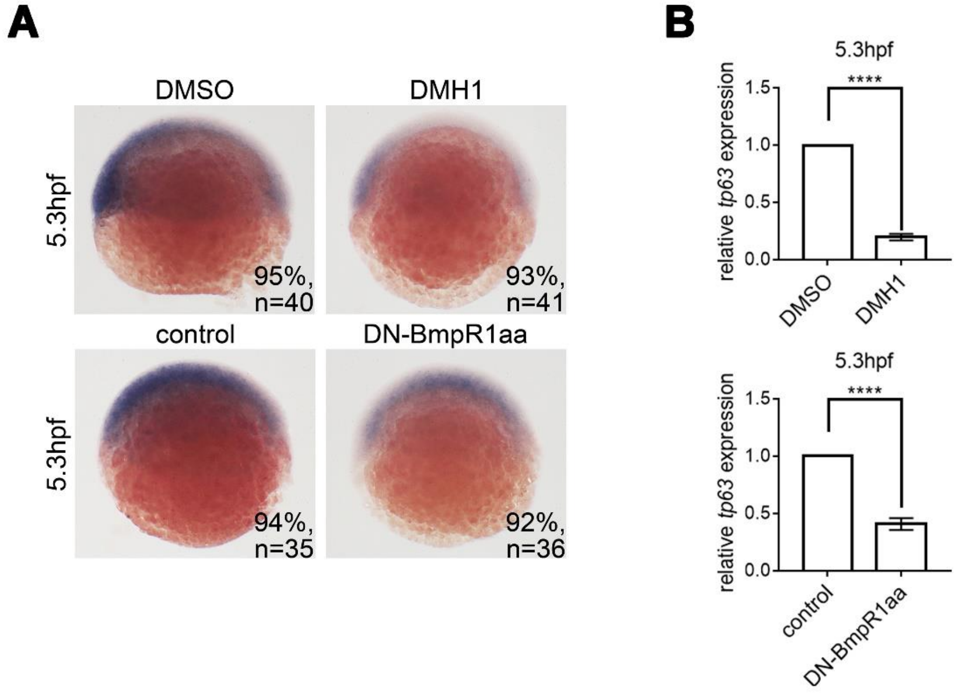

Figure S2. Blocking BMP signaling from 4 hpf inhibits the development of epidermal ectoderm.

(A) *In situ* hybridization using the *tp63* riboprobe. Lateral views, ventral to the left. At 5.3 hpf, *tp63* is expressed in the ventral part of the animal pole, which is the presumptive epidermal ectoderm. Inactivation of BMP signaling by heat-shock activation of DN-Bmpr1aa or DMH1 treatment from 4 hpf was sufficient to inhibit *tp63* expression in embryos harvested at 5.3 hpf. (B) *tp63* expression was quantified using qRT-PCR. The percentages in each panel in A indicate the proportion of embryos displaying the same phenotype as that shown in the photographs of the total embryos examined. Quantitative data are presented as mean ± standard deviation (SD). ****, $p < 0.0001$.

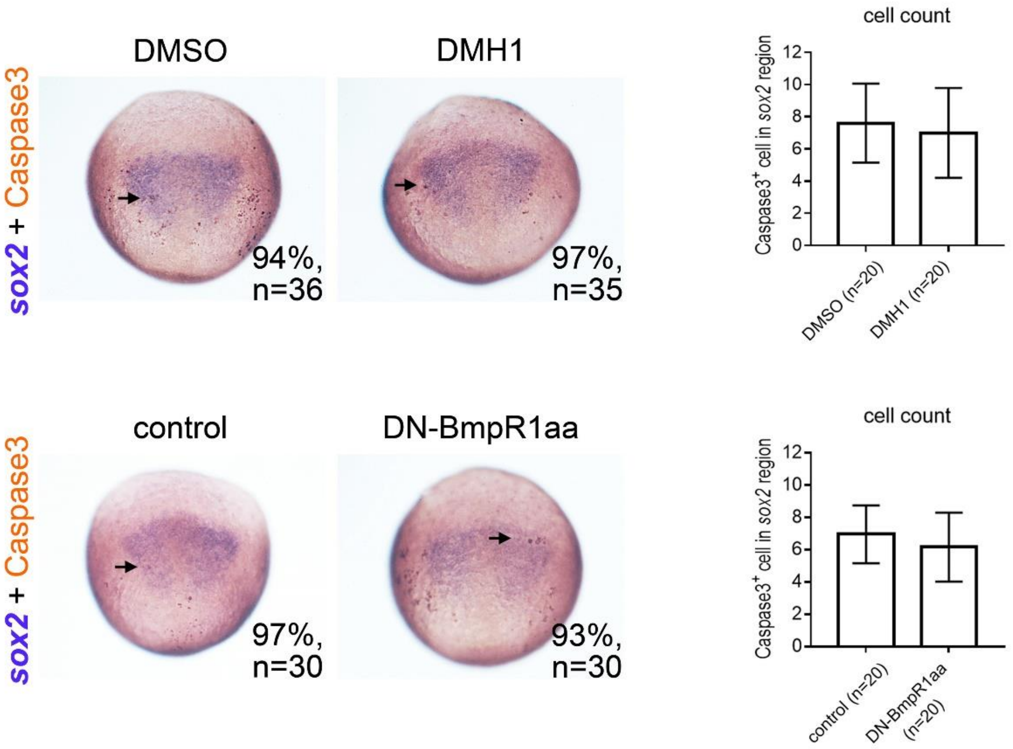

Figure S3. Inactivation of BMP-Smad signaling does not affect cell apoptosis. (Left panels) Embryos were harvested at 8 hpf and subjected to *in situ* hybridization with a *sox2* riboprobe and immunohistochemistry with an activated caspase-3 antibody (brown). The double-labeled cells are indicated with arrows. Inactivation of BMP-Smad signaling by DN-Bmpr1aa or DMH1 did not affect the number of *sox2* and caspase-3 double-positive cells. (Right panels) The number of caspase-3 positive cells in *sox2*-expressing neuroectoderm was counted manually, and the result demonstrated that the inactivation of BMP-Smad signaling did not alter the number of apoptotic cells. The percentages in each panel indicate the proportion of embryos displaying the same phenotype as that shown in the photographs of the total embryos examined. Values are presented as the mean ± SD.
